# Association of clinical biomarkers and response to neoadjuvant therapy in breast cancer

**DOI:** 10.1007/s11845-023-03489-1

**Published:** 2023-09-07

**Authors:** Gerard Feeney, Ronan Waldron, Nicola Miller, Carmel Malone, Karl Sweeney, Raymond McLaughlin, Aoife Lowery, Kevin Barry, Michael Kerin

**Affiliations:** 1 Lambe Institute for Translational Research, National University of Ireland, Galway, Ireland; 2 Department of General Surgery, Galway University Hospital, Newcastle, Galway, Ireland

**Keywords:** Breast cancer, Lymphocyte-CRP ratio, Neoadjuvant therapy, Neutrophil–lymphocyte ratio, Oncology, Surgery

## Abstract

**Introduction:**

Neoadjuvant therapy is an essential component of multimodality therapy for locally advanced breast adenocarcinoma (BC). Complete pathologic response (pCR) is a useful surrogate for long-term oncologic outcome.

**Aim:**

To assess the association between clinicopathologic, molecular and immunological markers and treatment response to neoadjuvant therapy in BC.

**Methods:**

BC patients undergoing neoadjuvant therapy were identified from a prospectively maintained institutional database. Serum haematological/biochemical values, histopathologic, immunohistochemical data and TNM stage were obtained from electronic records. Patients were categorised into complete responders vs non-complete responders and responders vs non-responders. Statistical analysis was performed via SPSS.

**Results:**

Overall, 299 BC patients were included. The average age was 49.8 ± 11.5 years. A pCR was evident in 22.6% (*n* = 69). pCR was associated with early T stage and non-luminal subtypes (HER2 enriched [HER2 +] and triple negative [TNBC]). The neutrophil–lymphocyte ratio (NLR) pre-operatively was lower in patients with a pCR (*p* = 0.02). The lymphocyte-CRP ratio (LCR) was also slightly reduced in responders (*p* = 0.049) at diagnosis. A pre-op NLR greater than 2 was not found to be a significant predictive factor (p = 0.071) on multivariable logistic regression analysis. T stage at diagnosis (*p* = 0.024), N stage (*p* = 0.001) and breast cancer subtype (*p* = 0.0001) were also determined to be significant predictive factors of complete response.

**Conclusion:**

pCR was more likely in patients with less advanced disease in BC. The presence of HER2 + or TNBC in BC also increases the likelihood of pCR. Neoadjuvant therapy stimulates the systemic inflammatory response; however, a reduced baseline NLR may be associated with increased pCR. Confirmation with larger datasets is required.

## Introduction

The relationship between the tumour microenvironment and the host immune system has been recognised as an essential component in tumourigenesis. A comprehensive understanding of these interactions provides insight into both the propagation of tumour development and potential therapeutic targets. Recognition of the tumour by the host immune system results in the initiation of a pro-inflammatory state, secondary to cytokine and chemokine secretion. This allows for the recruitment of lymphocytes, particularly CD8 + cytotoxic lymphocytes, to infiltrate the tumour stroma and eliminate tumour cells. The function of these cytotoxic cells is augmented through natural killer (NK) cells and inhibited through the action of CD4 + regulatory lymphocytes. Several studies have demonstrated a positive association between tumour burden and degree of systemic inflammation within the host [1–3].

In order to maintain proliferation, tumour cells seek to inactivate or neutralise the action of CD8 + T cells. Thus, cancer patients frequently demonstrate altered levels of immune cells in the peripheral circulation. For instance, breast tumours have been noted to promote the development of neutrophilia in patients. Increases in circulating neutrophils serves to minimise the tumour surveillance activity of CD8 + T cells, thus promoting the development of breast carcinoma [4]. Breast cancer patients with lymphopaenia secondary to malignancy have demonstrated poorer outcomes, in relation to recurrence and mortality.

Analysis of the relationship between the level of circulating immune cells and tumours is typically expressed in the form of ratios of specific immune cells or inflammatory markers. For instance, neutrophil–lymphocyte ratios (NLR) have been investigated extensively in breast cancer [5]. Studies have demonstrated poor outcomes in breast cancer patients with higher NLR values [5]. This is secondary to an increased systemic inflammatory response, represented as neutrophilia, in conjunction with reduced tumour clearance capacity, represented through lymphopaenia. Similar ratios have also been assessed in other cancer states. For example, the lymphocyte-CRP ratio (LCR) was analysed in colorectal cancer outcomes [6]. The authors noted improved outcomes in patients with elevated LCR. In this particular ratio, CRP serves as a surrogate marker for systemic inflammation. To date, there has been no published data on the relationship between breast cancer and LCR values. Additionally, circulating monocytes have been documented to have anti-tumoural effects through the promotion of lymphocyte recruitment and differentiation of monocytes into tumour-associated macrophages [7]. Thus, studies investigating breast cancer outcomes with lymphocyte-monocyte ratio (LMR) expression have determined a positive relationship between improved survival and high LMR values [8]. These ratios and their resultant clinical outcomes are summarised in Tables 1, 2, 3 and 4.

**Table 1:** Immunological ratios in breast cancer

Ratio	Increased/decreased in cancer states	Impact of therapy on expression	Cutoff values
NLR	↑ [10]	↓ [11]	2.21 (breast Ca) [11]
LCR	↓ [6]	N/A	6000 (rectal Ca) [6]
LMR	↓ [8]	↑ [12]	5.46 (breast Ca) [12]

The role of neoadjuvant therapy in cancer therapy is to promote the development of local inflammation at the site of the tumour and, thus, enhance migration and recruitment of lymphocytes for the purpose of minimising tumour burden. Conversely, neoadjuvant chemotherapy may also result in activation of a systemic inflammatory response, which entails a wide range of adverse effects for patients, including, neutropaenic sepsis, deep venous thrombosis/pulmonary embolus, mucositis and so on. Therefore, the goal of neoadjuvant chemotherapy is to maximise local inflammation and minimise systemic inflammation. The ideal result of neoadjuvant therapy is the attainment of complete pathological response (PCR). Upon analysis of histopathological resection specimens, PCR was observed in 19% of breast cancer patients [9]. Significant focus has been placed on the elucidation of predictive models towards which patients are likely to derive PCR from neoadjuvant therapy. The utilisation of easily measured inflammatory markers in the peripheral circulation may be of benefit in the construction of such a clinical model.

Therefore, the aim of this study is to assess the expression of circulating inflammatory markers in breast cancer patients who received neoadjuvant chemotherapy to determine potential as a clinical biomarker. Analysis will be made of the relationship between ratios of inflammatory markers and patient outcomes, in particular, response rates to neoadjuvant therapy.

## Methods

Overall, 299 breast cancer patients were included in this study. All patients had histologically proven breast adenocarcinoma. Patients of all breast cancer subtypes and histology were included. Neoadjuvant therapy was prescribed in all cases following discussion at the Multidisciplinary Meeting and surgical resection were performed with curative intent. All patients were treated at Galway University Hospital between 2009 and 2017. Palliative patients were excluded from the study. In addition, patients with incomplete biochemical data were also excluded. Diagnostic and therapeutic clinicopathological data was obtained from prospectively maintained databases of breast cancer patients treated surgically at our institution. Datapoints included patient age, gender, procedure performed and staging information. Neoadjuvant regimes were determined following interrogation of the local MOSAIQ and PAS software.

Haematological and biochemical data was obtained from electronic records. Three specific timepoints were assessed: diagnosis, pre-operative and post-operative. Diagnosis was defined as the date of the confirmatory biopsy. Pre-operative was the sample obtained immediately preceding surgical resection and following completion of neoadjuvant therapy. Post-operative values were routinely recorded no less than 3–5 days post-procedure. These specific timepoints were chosen to highlight the potential impact of neoadjuvant therapy (∆diagnosis-preoperative) and surgical therapy (∆preoperative-postoperative) on each individual ratio. Patients with incomplete or missing haematological data were not included in the study analysis.

Response to neoadjuvant therapy was determined from histopathology reports signed off by Consultant Histopathologists. Complete response was defined as specimens with no residual tumour identified. Responders were defined as specimens that demonstrated some measure of fibrous tissue in place of tumour tissue. No response was classified as the absence of fibrous tissue in the resected specimen or the presence of more advanced disease compared to clinical staging. Local recurrence information was gathered from radiology records. The date of local recurrence was defined as the same date as a confirmatory scan was performed. Mortality data was elicited from local MOSAIQ and PAS software. All survival data was up to May 2020.

Statistical analysis was performed on SPSS Version 23. Chi-square and Fishers exact test was conducted to assess crosstabulations. Mann–Whitney *U* and Kruskal–Wallis tests were used to investigate non-parametric data. Students *t*-test and ANOVA were utilised to analyse parametric data. ROC curves were generated to determine appropriate cutoff values for each ratio. Associations with treatment response were assessed with multivariable and univariable logistic regression analysis. Associations with survival were performed with Cox regression hazard models. A *p* value of less than 0.05 was considered statistically significant in all tests.

**Table 2:** Number of samples analysed in breast and rectal cancer cohorts

	Diagnostic samples (*n* =)	Pre-operative samples (*n* =)	Post-operative samples (*n* =)	Total samples (*n* =)
Breast cancer	299	299	299	897

## Results

Within the breast cancer cohort, the mean age of patients was 49.8. Invasive ductal carcinoma occurred in 216 (70.8%) of patients in this cohort with luminal A being the most frequent breast cancer subtype (*n* = 144, 47.2%). HER2-enriched cancers accounted for 45 (14.7%) cases with triple negative subtype identified in 59 (19.3%). The majority of patients presented with T2 disease at diagnosis (*n* = 105, 34.4%) with 211 (70.8%) also presenting with some form of nodal disease (*N* ≤ 1) at the time of diagnosis.

The most common surgical procedure performed was the mastectomy (*n* = 164, 53.8%) with breast conserving surgery undertaken in the remaining 134 cases (43.9%). Following analysis of the resected surgical specimen, complete pathological response (pCR) was observed in 69 (22.6%) with downstaging noted in a further 95 (31.8%). The remainder (*n* = 134, 43.9%) demonstrated no response to neoadjuvant therapy. The mortality rate at 5 years was 12.8% (*n* = 39). Local recurrence occurred in 28 (9.2%) patients after a follow-up period of 5 years. Development of distant metastases was present in 30 (9.8%) at 5 years post-procedure.

**Table 3:** Patient characteristics

		***N* = (%)**
Overall		299
Average age	Mean ± standard deviation	49.8 ± 11.5
Presentation	Symptomatic	284 (95.3%)
	Screening	14 (4.7%)
Histology	Ductal	216 (70.8%)
	Lobular	25 (8.2%)
	Inflammatory	28 (9.2%)
	Other	20 (6.6%)
Subtype	Luminal A	144 (47.2%)
	Luminal B	50 (16.4%)
	Triple negative	59 (19.3%)
	HER2	45 (14.7%)
T stage	1	27 (8.9%)
	2	105 (34.4%)
	3	54 (17.7%)
	4	35 (11.5%)
N stage	0	46 (15.1%)
	1	149 (48.9%)
	2	47 (15.4%)
	3	15 (4.9%)
Tumour grade	0	12 (3.9%)
	1	7 (2.3%)
	2	139 (45.6%)
	3	141 (46.2%)
DCIS	Present	146 (47.9%)
	Absent	158 (52.1%)
Neoadjuvant chemotherapeutic agents	AC w/paclitaxel	148 (49.7%)
	TC	86 (28.8%)
	Carboplatin	7 (2.3%)
	Other	6 (2%)
Neoadjuvant therapy cycles	1	4 (1.3%)
	2	1 (0.3%)
	3	4 (1.3%)
	4	169 (55.4%)
	5	2 (0.7%)
	6	60 (19.7%)
Treatment response:	Complete	69 (22.6%)
	Downstaging	95 (31.8%)
	No response	134 (43.9%)
Surgical procedure	Breast conserving surgery	134 (43.9%)
	Mastectomy	164 (53.8%)
Total nodes harvested	Mean ± standard deviation	14.2 ± 8.7
Total positive nodes	Mean ± standard deviation	2.87 ± 5.1
Sentinel nodes harvested	Mean ± standard deviation	3.9 ± 3.1
Positive sentinel nodes	Mean ± standard deviation	0.65 ± 1.1
Mortality status @ 5 years	Alive	256 (83.9%)
	RIP	39 (12.8%)
Recurrence status	None	237 (77.7%)
	Local	28 (9.2%)
	Distant	30 (9.8%)
CA 15-3	Diagnosis	30.75 ± 23.9
	Pre-operatively	35.54 ± 18.4
	Post-operatively	27.48 ± 43.2
Neutrophil-lymphocyte ratio	Diagnosis	3.3 ± 2.6
	Pre-operatively	3.4 ± 2.4
	Post-operatively	4.1 ± 2.8
Lymphocyte-CRP ratio	Diagnosis	1.9 ± 1.3
	Pre-operatively	0.9 ± .7
	Post-operatively	0.58 ± .7
Platelet-lymphocyte ratio	Diagnosis	202 ± 138.4
	Pre-operatively	250.2 ± 132.3
	Post-operatively	255.8 ± 119
Lymphocyte-monocyte ratio	Diagnosis	5.1 ± 2.9
	Pre-operatively	3.9 ± 2.1
	Post-operatively	4 ± 2.7

**Table 4:** Complete responders vs. non-complete responders crosstabulation

		**Responders (*N* = 164)**	**Non-responders (*N* = 134)**	***p* value**	**Complete response (*N* = 69)**	**Non-complete response (*N* = 229)**	***p* value**
**Subtype**	Luminal A	44 (26.8%)	81 (60.4%)	***0.0001***	9 (13%)	134 (58.5%)	***0.0001***
	Luminal B	28 (17.1%)	21 (15.7%)		15 (21.7%)	35 (15.3%)	
	Triple Negative	38 (23.2%)	15 (11.2%)		25 (36.2%)	34 (14.8%)	
	HER2	29 (17.7%)	15 (11.2%)		20 (29%)	24 (10.5%)	
**T stage**	1	9 (5.5%)	18 (13.4%)	***0.0001***	8 (11.6%)	19 (8.3%)	0.094
	2	50 (30.5%)	55 (41%)		22 (32.9%)	83 (36.2%)	
	3	15 (9.1%)	39 (29.1%)		7 (10.1%)	47 (20.5%)	
	4	31 (18.9%)	4 (3%)		2 (1.9%)	33 (14.4%)	
**N stage**	0	17 (10.4%)	24 (17.9%)	0.34	10 (14.5%)	36 (15.7%)	***0.0001***
	1	73 (44.5%)	65 (48.5%)		46 (66.7%)	103 (45%)	
	2	23 (14%)	20 (14.9%)		0	47 (20.5%)	
	3	5 (3%)	11 (8.2%)		0	15 (6.6%)	
**Tumour grade**	0	8 (4.9%)	3 (2.2%)	***0.006***	6 (8.7%)	5 (2.2%)	***0.006***
	1	1 (0.6%)	5 (3.7%)		0	7 (3.1%)	
	2	55 (33.5%)	75 (55.9%)		24 (34.8%)	115 (50.2%)	
	3	75 (45.7%)	51 (38.1%)		39 (56.5%)	102 (44.5%)	
**Histology**	Ductal	93 (56.7%)	105 (78.4%)	***0.001***	56 (81.2%)	160 (69.9%)	***0.013***
	Lobular	6 (3.7%)	17 (12.7%)		0	25 (10.9%)	
	Inflammatory	24 (14.6%)	1 (0.7%)		3 (4.3%)	23 (10%)	
	Other	8 (4.9%)	8 (6%)		4 (5.8%)	16 (7%)	
**Recurrence**	None	116 (70.7%)	102 (76.1%)	0.107	62 (89.9%)	175 (76.4%)	***0.011***
	Local	12 (7.3%)	13 (9.7%)		1 (1.4%)	27 (11.8%)	
	Distant	9 (5.5%)	19 (14.2%)		4 (5.8%)	26 (11.4%)	
**Ductal in situ**	Present	77 (47%)	59 (44%)	0.113	21 (30.4%)	63 (27.5%)	***0.009***
	Absent	31 (18.9%)	38 (28.4%)		17 (24.6%)	129 (56.3%)	
**Surgical procedure performed**	Mastectomy	77 (47%)	74 (55.2%)	0.483	29 (42%)	135 (59%)	***0.013***
	BCS	57 (34.8%)	65 (48.5%)		40 (56%)	94 (41%)	
**5-year mortality**	RIP	20 (12.2%)	17 (12.7%)	0.546	1(1.4%)	38 (16.6%)	***0.001***
	Alive	114 (69.5%)	120 (89.6%)		66 (95.7%)	190 (83%)	

### Response to NA therapy

Overall, 164 patients (71.6%) demonstrated a degree of significant response to therapy, either complete response or downstaging. This particular subgroup also demonstrated that response was more likely in those with non-luminal breast cancer (*p* = 0.001). A measure of response to therapy was more apparent in those with less advanced disease also (T stage: *p* = 0.0001, tumour grade: *p* = 0.06).

The rate of pCR in the breast cancer cohort was 30.1% (*n* = 69). Upon analysing this subgroup, 45 (65.2%) were non-luminal breast cancers with Her2-enriched subtype representing *n* = 20 (29%) and triple negative contributing *n* = 25 (36.2%) (i = 0.0001). Complete responders did not demonstrate any advanced nodal disease at diagnosis (> N1) (*p* = 0.001). Patients who underwent breast conserving surgery were more likely to derive a complete response compared to those with mastectomy (*p* = 0.013). Patients who derived a complete response from NA therapy were less likely to develop a recurrence, either local or distant, during their 5-year follow-up period (*p* = 0.011). Complete response was also related to improved survival outcomes with only 1 mortality event occurring amongst complete responders at 5 years (*p* = 0.001).

### Systemic inflammatory ratios

NLR demonstrated a consistent increase throughout the course of breast cancer therapy. The average NLR increased from 3.3 at diagnosis to 4.1 in the post-operative period (*p* = 0.0001).

In contrast to NLR, average LCR values demonstrate a consistent decline upon the treatment of breast cancer. Average LCR values reduce from 1.9 at diagnosis to 0.9 preceding surgery and 0.6 following surgical treatment (*p* = 0.0001).

**Fig. 1:**
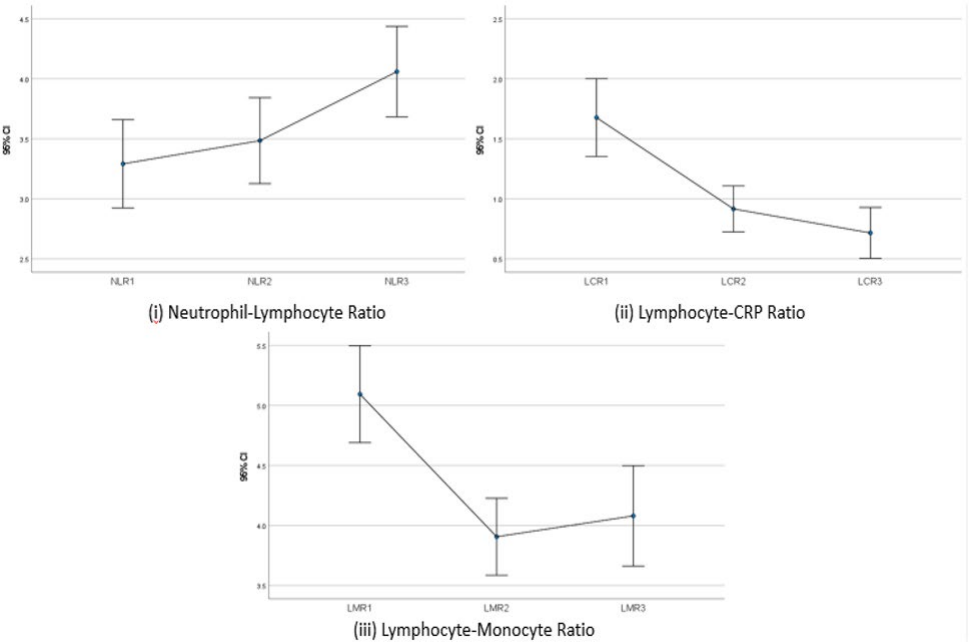
Impact of breast cancer therapy on NLR, LCR and LMR (1 = diagnosis, 2 = pre-operative, 3 = post-operative) ((i) NLR demonstrated consistent increase throughout treatment; (ii) LCR was downregulated over the course of treatment; (iii) LMR decreased during neoadjuvant therapy, with an increase post-surgery)

**Fig. 2:**
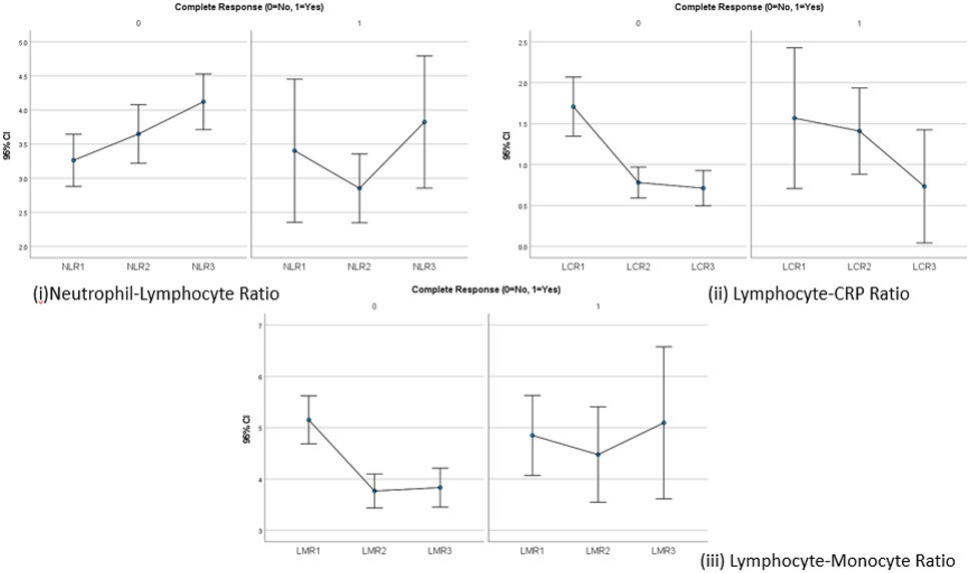
NLR, LCR and LMR in complete responders vs. non-complete responders in breast cancer (1 = diagnosis, 2 = pre-operative, 3 = post-operative) ((i) NLR at pre-operative significantly lower in complete responders (*p* = 0.027); (ii) average LCR values routinely higher in complete responders; (iii) average LMR lower at diagnosis in complete responders but greater in the pre- and post-operative periods)

**Fig. 3:**
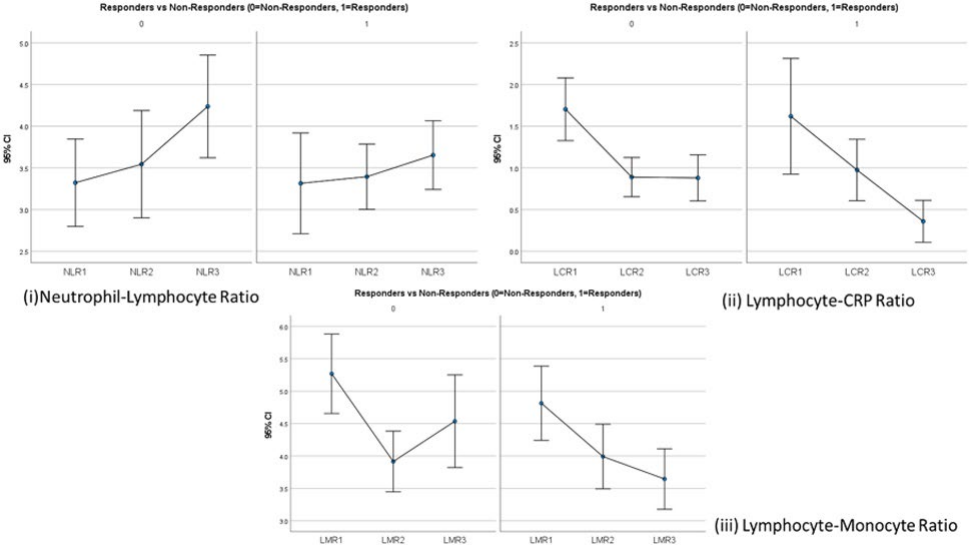
NLR, LCR and LMR in responders vs. non-responders in breast cancer (1 = diagnosis, 2 = pre-operative, 3 = post-operative) ((i) average NLR lower in responders; (ii) average LCR elevated at diagnosis in responders; (iii) average LMR lower at diagnosis and post-operatively in responders)

**Fig. 4:**
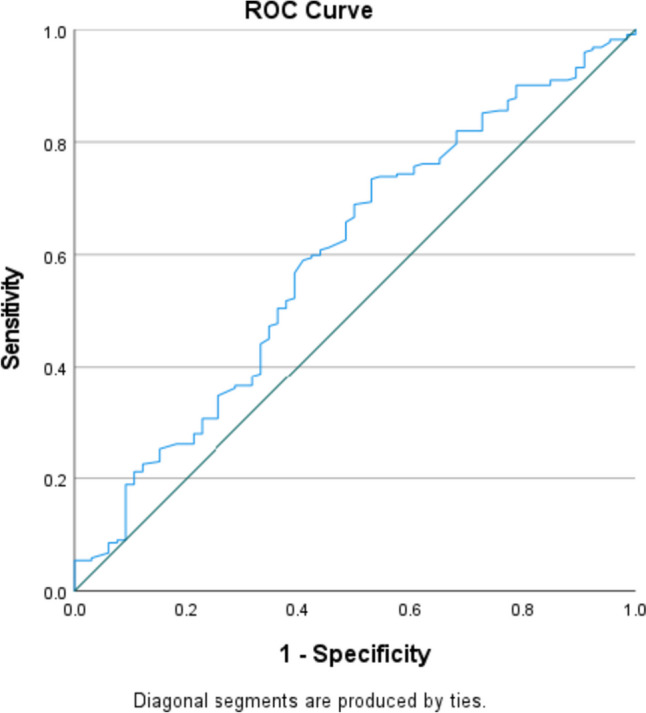
ROC curve for Pre-Op NLR predicting complete response

**Fig. 5:**
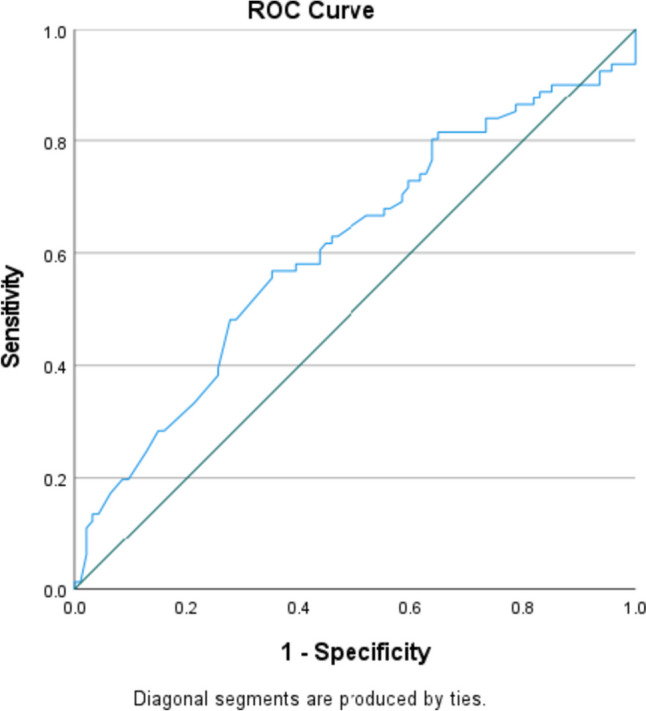
ROC curve for diagnostic LCR predicting treatment response

The lymphocyte-monocyte ratio was noted to decrease in breast cancer patients following neoadjuvant therapy from 5.1 at diagnosis to 3.9 post-neoadjuvant therapy (*p* = 0.0001). Following surgical resection, LMR was observed to increase, to an average value of 4 indicating a reduction in systemic inflammation upon definitive treatment of the tumour (Figs. 1, 2, 3, 4 and 5).

### Systemic inflammatory markers and treatment response

The overall trend of increasing NLR through therapy was broadly replicated when comparing complete responders vs. non-complete responders in the breast cancer cohort. The average NLR at diagnosis was 3.6 and 3.2 for complete responders and non-complete responders, respectively (*p* = 0.89). The same values at the pre-operative timepoint were 2.9 and 3.6, respectively (*p* = 0.02). The median NLR for complete responders post-operatively was 4 and 4.1, respectively (*p* = 0.34).

LCR exhibited declines in both complete responders and non-complete responders over the course of breast cancer therapy. LCR values were noted to be higher in complete responders at diagnosis (2.2 vs. 1.8, *p* = 0.149) and in the pre-operative period (1.2 vs 0.8, *p* = 0.216). LCR was elevated amongst complete responders post-operatively (0.56 vs. 0.59, *p* = 0.852). None of these differences was statistically significant.

In patients who derived a complete response from neoadjuvant therapy, LMR was observed to be lower at diagnosis (4.7 vs 5.2, *p* = 0.726) but higher in the remaining two timepoints (4.2 vs. 3.9 pre-operatively, 4.4 vs. 3.9 post-operatively). None of these differences was statistically significant. Both cohorts demonstrated a similar trend of a decrease in LMR following neoadjuvant therapy and subsequent LMR elevation after surgery.

Upon comparison of responders vs. non-responders in breast cancer patients, NLR demonstrated a consistent increase over the duration of treatment similar to the overall patient population. NLR at diagnosis was 3.4 and 3.3 for responders and non-responders, respectively (*p* = 0.893). At the pre-operative period, NLR was 3.6 and 3.4, respectively (*p* = 0.266).

On considering LCR differences between responders and non-responders, average LCR was greater in responders at diagnosis (2.1 vs. 1.6, *p* = 0.014). LCR was lower in responders at the pre-operative (0.9 vs. 0.99, *p* = 0.29) and post-operative timepoints (0.5 vs. 0.7, *p* = 0.217). As in complete responders, none of these differences was statistically significant.

Identical trends to complete responders were identified when LMR was assessed between responders and non-responders. Average LMR was reduced in responders at diagnosis (4.9 vs. 5.3, *p* = 0.543) and post-operatively (3.7 vs. 4.5, *p* = 0.075) with responders demonstrating a more elevated LMR in the pre-operative (4.1 vs. 4, *p* = 0.906) period. Similar to the complete responders, none of the differences was statistically significant.

### ROC curve analysis

ROC curve analysis for predicting the sensitivity and specificity of pre-operative NLR regarding complete response generated an area under the curve (AUC) value of 0.594. Utilising a Pre-Op NLR value of 2 generated a sensitivity of 77% and 1-specificity value of 65.2%.

A cutoff value of 2 was chosen for pre-op NLR and a multivariable logistic regression analysis was performed to determine independent prognostic factors for complete response to neoadjuvant therapy in breast cancer. A pre-op NLR greater than 2 was not found to be a significant predictive factor (*p* = 0.071). T stage at diagnosis (*p* = 0.019), N stage (*p* = 0.003), tumour grade (*p* = 0.024), histological type (*p* = 0.019) and breast cancer subtype (*p* = 0.0001) were determined to be significant predictive factors of complete response. T stage at diagnosis (*p* = 0.006), N stage (*p* = 0.001) and breast cancer subtype (*p* = 0.001) remained significant independent predictive factors of complete response on univariate logistic regression analysis.

ROC curve analysis for predicting the sensitivity and specificity of diagnostic LCR regarding therapy response generated an area under the curve (AUC) value of 0.607. Utilising a diagnostic LCR value of 1.87 generated a sensitivity of 58% and 1-specificity value of 39.4%.

A cutoff value of 1.87 was chosen for diagnostic LCR and a multivariable logistic regression analysis was performed to determine independent prognostic factors for treatment response to neoadjuvant therapy in breast cancer. A diagnostic LCR of less than 1.87 was not found to be a significant predictive factor of treatment response (*p* = 0.098). T stage at diagnosis (*p* = 0.0001), histological type (*p* = 0.0001) and breast cancer subtype (*p* = 0.002) were determined to be significant predictive factors of treatment response. T stage at diagnosis (*p* = 0.007), invasive histological type (*p* = 0.016) and breast cancer subtype (*p* = 0.0001) remained significant independent predictive factors of treatment response on univariate logistic regression analysis.

## Discussion

This study has demonstrated the intrinsic relationship between the systemic immune response of the host and the clinical behaviour and outcomes of the tumour. In particular, our results demonstrate that patients with a reduced systemic inflammatory response to neoadjuvant therapy have higher rates of response to this therapy. Response to neoadjuvant therapy has been well recognised as a predictor for improved long-term outcomes in breast cancer patients. Our findings are in keeping with previously published literature assessing the systemic immune system in breast cancer patients [13]. Initial examination of outcomes in relation to clinicopathological parameters highlighted that patients with more advanced disease were less likely to derive a benefit from neoadjuvant chemotherapy. We also observed an inverse association between the presence of nodal metastases and the incidence of complete response. Likewise, an investigation of 13,396 patients by Kantor et al. found that breast cancer patients with nodal disease burden were less likely to derive a response from neoadjuvant therapy [14].

The same study also observed that triple negative and HER2-positive tumours were more responsive to neoadjuvant chemotherapy. Our findings also indicated that non-luminal breast cancers were more chemosensitive. Numerous studies have demonstrated the rarity with which luminal breast cancers achieve pCR [15, 16]. Triple negative breast cancers (TNBC) have been shown to have greater chemosensitivity compared to other breast cancer subtypes. A study by Liedtke et al. reported a higher pCR for TNBC patients relative to other subtypes (22% vs. 11%, *p* = 0.034). TNBC patients with pCR proceeded to have similar survival outcomes in comparison with other subtypes [15]. PCR in breast cancer has been proven to confer improved survival outcomes on breast cancer patients. The Collaborative Trial on Neoadjuvant Breast Cancer examined data from 12 trials and 11,955 patients investigating long-term outcomes in those with pCR. The authors found that pCR was associated with superior overall survival and that this effect was most pronounced in the high-risk subtypes (HER2 and TNBC) [17].

Our data delineates the gradual increase in systemic inflammation throughout the treatment pathway for breast cancer patients. This is secondary to neoadjuvant and surgical therapy of their disease. Cancer often results in chronic inflammation which is manifested by a systemic neutrophilia [18]. Neutrophils have been documented to play an integral role in the secretion of pro-inflammatory cytokines and deactivation of surveillance T cells, thus, propagating the tumour-mediated chronic inflammatory process [4, 19]. Consequently, neutrophilia in turn results in lymphopaenia, thus, resulting in elevated NLR in cancer patients.

From this information, we may deduce that breast cancer patients with relatively high NLR values will experience poorer outcomes, specifically in this instance, poor therapeutic response. From our data, patients who demonstrate some form of response to therapy, have a lower average NLR value. In patients with breast cancer, our data has shown a significant decrease in average NLR between complete responders and non-complete responders. These findings have been replicated in other similar studies [13, 20, 21]. Reduced NLR at diagnosis was observed by Chen et al. to be predictive of pCR in a cohort of 215 breast cancer patients [13]. Furthermore, low baseline NLR was related to improved recurrence-free survival in the same study. NLR has also demonstrated sensitivity in predicting pCR amongst TNBC patients [20]. Asano et al. investigated potential correlations between baseline NLR and outcomes in 177 breast cancer patients. The authors found that a low NLR was predictive of pCR with TNBC patients being the most frequent complete responders.

Lymphocyte-CRP ratio takes into account both the adaptive immune system and systemic inflammation, represented as lymphocyte count and CRP, respectively. Lymphocytes, in particular CD8 + T cells are essential in mediating the adaptive immune response to invasive tumour cells [22]. Ideally, the immune response and subsequent inflammation will be localised to the tumour and inflammation will not become disseminated throughout the host. Analysis of numerous rectal tumour biopsies noted that the rate of therapy response was proportional upon the level of CD8 + tumour infiltrating lymphocytes (TILs) [22]. Increased systemic inflammation, in the form of elevated CRP, indicates a systemic inflammatory response which is indicative of both advanced disease and impaired local control of inflammation. Thus, the LCR should be reduced in those with sufficient immune control and elevated in patients with poor localised control. Our data demonstrated a lower average LCR in patients who responded to neoadjuvant therapy, in breast cancer. No studies have been performed investigating LCR in breast cancer to date.

The lymphocyte-monocyte ratio (LMR) acts as a further assessment of the hosts systemic inflammatory response by measuring the proportion of lymphocytes and monocytes in the peripheral circulation. Within the immune system, monocytes serve to activate and recruit lymphocytes for the purpose of eliminating foreign antigens [7]. The LMR has previously been assessed in other cancers, for example, pancreatic [23] and oesophageal cancer [24]. Results have been conflicting with elevated LMR regarded as a positive prognostic factor in pancreatic cancer and low LMR seen as beneficial in long-term outcomes for upper GI cancers.

In our study, the relationship between LMR values and therapy response have been mixed. In patients who derived pCR, average LMR was reduced particularly, at the pre-operative timepoint. These findings are in contradiction to those elucidated by Goto et al., whereby low LMR was found to confer poor long-term outcomes in 239 patients analysed [8]. This was most pronounced in TNBC patients (*p* = 0.006). Another study by Ma et al. similarly related low average LMR values with poor long-term outcomes in breast cancer [25].

There are several limitations to this study. The study design was retrospective in nature. Secondary to this, some elements of clinicopathological data from individual patients were unable to be retrieved adequately and were, thus, omitted from this study. This was particularly the case in instances where patients had been treated at a time preceding the introduction of comprehensive electronic records in our institution. The sample size was small, and thus, significance was unable to be achieved in logistic regression analysis. The sample size also impacted upon the ROC curve analysis with the generation of low sensitivity values for specific ratios. It is possible that further studies with larger sample sizes may overcome these limitations. Another challenge in data collection was the relative paucity of CRP tests performed on breast cancer patients at diagnosis. This led to the exclusion of numerous patients whose baseline LCR could not be calculated, further minimising the available sample size.

Conversely, this study also has several strengths. Data obtained from this study was from a prospectively maintained database. All patients were diagnosed and treated from a single centre and, thus, every patient underwent identical multidisciplinary and decision-making processes. This provided for continuity of care and consistent treatment decisions being made in a collaborative setting amongst different oncological specialties.

In conclusion, this study has investigated the systemic immune profile of breast cancer patients who have undergone neoadjuvant therapy. From this data, we have found significant positive associations between pCR and both non-luminal subtype and nodal metastases in breast cancer. Furthermore, we have described the increase in systemic inflammation over the course of treatment. We have found that, in breast cancer, pre-operative NLR is significantly reduced in pCR, indicating reduced levels of systemic inflammation in patients who derive a positive benefit from neoadjuvant therapy. These results support the theory that systemic inflammation is affected by cancer treatment and that an exaggerated systemic inflammatory response is deleterious to the effects of neoadjuvant therapy and by extension, long-term outcomes. Further studies with larger sample sizes are required to validate these results.

## Data Availability

The data presented in this study are available on request from the corresponding author. The data are not publicly available due to patient confidentiality records.

## References

[1] Grivennikov SI, Greten FR, Karin M (2010). Immunity, inflammation, and cancer. Cell.

[2] Fernandes JV, Cobucci RN, Jatobá CA (2015). The role of the mediators of inflammation in cancer development. Pathol Oncol Res : POR.

[3] Elinav E, Nowarski R, Thaiss CA (2013). Inflammation-induced cancer: crosstalk between tumours, immune cells and microorganisms. Nat Rev Cancer.

[4] Noh H, Eomm M, Han A (2013). Usefulness of pretreatment neutrophil to lymphocyte ratio in predicting disease-specific survival in breast cancer patients. J Breast Cancer.

[5] Ethier JL, Desautels D, Templeton A (2017). Prognostic role of neutrophil-to-lymphocyte ratio in breast cancer: a systematic review and meta-analysis. Breast Cancer Res : BCR.

[6] Okugawa Y, Toiyama Y, Yamamoto A (2020). Lymphocyte-C-reactive protein ratio as promising new marker for predicting surgical and oncological outcomes in colorectal cancer. Ann Surg.

[7] Olingy CE, Dinh HQ, Hedrick CC (2019). Monocyte heterogeneity and functions in cancer. J Leukoc Biol.

[8] Goto W, Kashiwagi S, Asano Y (2018). Predictive value of lymphocyte-to-monocyte ratio in the preoperative setting for progression of patients with breast cancer. BMC Cancer.

[9] Haque W, Verma V, Hatch S (2018). Response rates and pathologic complete response by breast cancer molecular subtype following neoadjuvant chemotherapy. Breast Cancer Res Treat.

[10] Faria SS, Fernandes PC, Silva MJ (2016). The neutrophil-to-lymphocyte ratio: a narrative review. Ecancermedicalscience.

[11] Kim HY, Kim TH, Yoon HK, Lee A (2019). The role of neutrophil-lymphocyte ratio and platelet-lymphocyte ratio in predicting neoadjuvant chemotherapy response in breast cancer. J Breast Cancer.

[12] Marín Hernández C, Piñero Madrona A, Gil Vázquez PJ (2018). Usefulness of lymphocyte-to-monocyte, neutrophil-to-monocyte and neutrophil-to-lymphocyte ratios as prognostic markers in breast cancer patients treated with neoadjuvant chemotherapy. Clin Transl Oncol : official publication of the Federation of Spanish Oncology Societies and of the National Cancer Institute of Mexico.

[13] Chen Y, Chen K, Xiao X (2016). Pretreatment neutrophil-to-lymphocyte ratio is correlated with response to neoadjuvant chemotherapy as an independent prognostic indicator in breast cancer patients: a retrospective study. BMC Cancer.

[14] Kantor O, Sipsy LM, Yao K, James TA (2018). A predictive model for axillary node pathologic complete response after neoadjuvant chemotherapy for breast cancer. Ann Surg Oncol.

[15] Liedtke C, Mazouni C, Hess KR (2008). Response to neoadjuvant therapy and long-term survival in patients with triple-negative breast cancer. J Clin Oncol.

[16] Bonadonna G, Valagussa P, Brambilla C, Ferrari L (1993). Preoperative chemotherapy in operable breast cancer. Lancet.

[17] Cortazar P, Zhang L, Untch M (2014). Pathological complete response and long-term clinical benefit in breast cancer: the CTNeoBC pooled analysis. Lancet.

[18] Howard R, Kanetsky PA, Egan KM (2019). Exploring the prognostic value of the neutrophil-to-lymphocyte ratio in cancer. Sci Rep.

[19] Coffelt SB, Kersten K, Doornebal CW (2015). IL-17-producing γδ T cells and neutrophils conspire to promote breast cancer metastasis. Nature.

[20] Asano Y, Kashiwagi S, Onoda N (2016). Predictive value of neutrophil/lymphocyte ratio for efficacy of preoperative chemotherapy in triple-negative breast cancer. Ann Surg Oncol.

[21] Chae S, Kang KM, Kim HJ (2018). Neutrophil–lymphocyte ratio predicts response to chemotherapy in triple-negative breast cancer. Curr Oncol.

[22] Huang Y, Lou XY, Zhu YX et al (2019) Local environment in biopsy better predict the pathological response to neoadjuvant chemoradiotherapy in rectal cancer. Biosci Rep 39(3) 10.1042/BSR20190003 PMC6434387 30867256

[23] Li W, Tao L, Zhang L, Xiu D (2017). Prognostic role of lymphocyte to monocyte ratio for patients with pancreatic cancer: a systematic review and meta-analysis. Onco Targets Ther.

[24] Huang Y, Feng J-F (2015). Low preoperative lymphocyte to monocyte ratio predicts poor cancer-specific survival in patients with esophageal squamous cell carcinoma. Onco Targets Ther.

[25] Ma Y, Zhang J, Chen X (2021). Lymphocyte-to-monocyte ratio is associated with the poor prognosis of breast cancer patients receiving neoadjuvant chemotherapy. Cancer Manag Res.

